# Bacterial contamination of mobile phones of health professionals in Eastern Ethiopia: antimicrobial susceptibility and associated factors

**DOI:** 10.1186/s41182-019-0144-y

**Published:** 2019-02-27

**Authors:** Dagne Bodena, Zelelam Teklemariam, Senthilkumar Balakrishnan, Tewodros Tesfa

**Affiliations:** 1 Hiwot Fana Specialized University Hospital, College of Health and Medical Sciences, Haramaya University, P.O. box 235, Harar, Ethiopia; 2 Department of Medical Laboratory Sciences, College of Health and Medical Sciences, Haramaya University, P.O. box 235, Harar, Ethiopia; 3 Department of Medical Microbiology, College of Health and Medical Sciences, Haramaya University, P.O. box 235, Harar, Ethiopia

**Keywords:** Bacteria, Health care professional, Mobile phone, Antimicrobial susceptibility, Hiwot Fana Specialized University Hospital, Eastern Ethiopia

## Abstract

**Background:**

Mobile phones of health care professionals could harbor microbes which cause nosocomial infections to the patient, family members, and the community at large. Thus, the aim of this study was to determine the prevalence of bacterial contamination of the mobile phones of health professionals, identify bacterial isolates, assess their antimicrobial susceptibility patterns, and define the associated factors.

**Method:**

A cross-sectional study was conducted from February to March 2018 on 226 health professionals’ mobile phones which were selected by a simple random sampling technique. Data were collected using a self-administered questionnaire. A swab sample from each of health professional’s mobile phone device was collected and transported to the microbiology laboratory for bacterial culture and antimicrobial susceptibility tests. Data were entered into EpiData version 3.1 and analyzed by using the Statistical Package for Social Sciences (SPSS) program version 20.

**Result:**

The overall prevalence of mobile phone contamination with one or more bacteria was 94.2%. Coagulase-negative staphylococci (CoNS; 58.8%), *Staphylococcus aureus* (14.4%), and *Klebsiella* species (6.9%) were the most predominant bacterial isolates. The overall prevalence of multidrug-resistant bacteria was 69.9%. About half of Gram-positive and Gram-negative bacteria were resistant to ampicillin and trimethoprim-sulfamethoxazole. Male sex (adjusted odds ratio (AOR) 4.1, 95% confidence interval (CI) 1.1, 15.8) and the absence of regular phone cleaning/disinfecting were found to be the most significant factors (AOR 4.1, 95% CI 1.2, 13.5) associated with health care professionals’ mobile phone bacterial contamination.

**Conclusion:**

There is a high contamination rate of mobile phones with nosocomial pathogens. Most of the isolates were resistant to ampicillin and trimethoprim-sulfamethoxazole and also multidrug-resistant. A mobile phone belonging to male health professionals and to those not disinfecting mobile phones was significantly contaminated with bacteria. Therefore, strategies for preventing nosocomial transmission of drug-resistant pathogens through mobile phones, like hand washing and cleaning mobile phones, are recommended.

## Background

A mobile phone is a long-range personal telecommunication device, easy to handle, and affordable to everybody [1]. It is the most indispensable accessory of professional and social life throughout the world [2]. Health care professionals’ mobile phones can be easily and quickly contaminated by microorganisms from the hospital environment, patients, and medical devices, since they use it for a medical dictionary, hand reference for drug, laboratory, and imaging results, and other work-related issues as they deal with patients having different illnesses [2–5]. Health care professionals constantly handle mobile phones without disinfection in their bags and pockets or on their hands in a clinical setup [6]. Patients are more vulnerable to nosocomial infections from a mobile phone which is often used near patients in hospital areas. Contaminated hands and mobile phones of health professionals can also play a great role in spreading infections to self, family member, and others outside the hospital [3–5].

There are some reports which indicate that giving low emphasis on regular disinfection of hands and poor hand washing practices by health professional predispose their and other individuals’ mobile phones to the colonization of bacteria [7, 8]. A study in the US revealed more than 80% of the common bacteria that make up our bacterial “fingerprints” end up on mobile phone screens [9].

Antimicrobial agents are used to controlling infection by susceptible pathogens. The emergence of antimicrobial resistance is associated with nosocomial infection, which is a serious public health problem. Some pathogens have become resistant to multiple drugs, and infections from resistant bacteria are now too common [10]. Drug resistance contributes substantially to the rising costs of health care, resulting from prolonged hospital stays and the need for more expensive and alternative drugs. These factors increase the stress of patients and their families as they face severe disability and reduce the patient’s quality of life [11, 12].

There are inconsistent reports on the contamination rate of a mobile phone of health professionals which indicate more than 80% of the phones were contaminated with different bacteria [6, 13–15], but there is no report from the eastern part of Ethiopia. Therefore, this study was aimed to assess the prevalence, antimicrobial susceptibility patterns, and factors associated with bacterial isolates from health professionals’ mobile phones working at Hiwot Fana Specialized University Hospital, Harar, Eastern Ethiopia.

## Materials and methods

### Study design, area, and period

A cross-sectional study was conducted on health professionals’ mobile phones working at Hiwot Fana Specialized University Hospital, Harar, Eastern Ethiopia, from February to March 15, 2018. Harar is located 526 km away from Addis Ababa, the capital city of Ethiopia. There are six hospitals (4 governments and 2 private hospitals), 8 health centers, and 26 health posts in the region. Hiwot Fana Specialized University Hospital (HFSUH) is one of the referral teaching hospitals in Ethiopia. Currently, the hospital provides health care service to more than five million peoples around Harar and neighboring regions like Oromiya Regional State, Dire Dawa Administrative Council, and Ethiopian Somali Regional State. The hospital has 787 workers in which 371 of these workers are health professionals.

### Sample size and sampling techniques

**Fig. 1:**
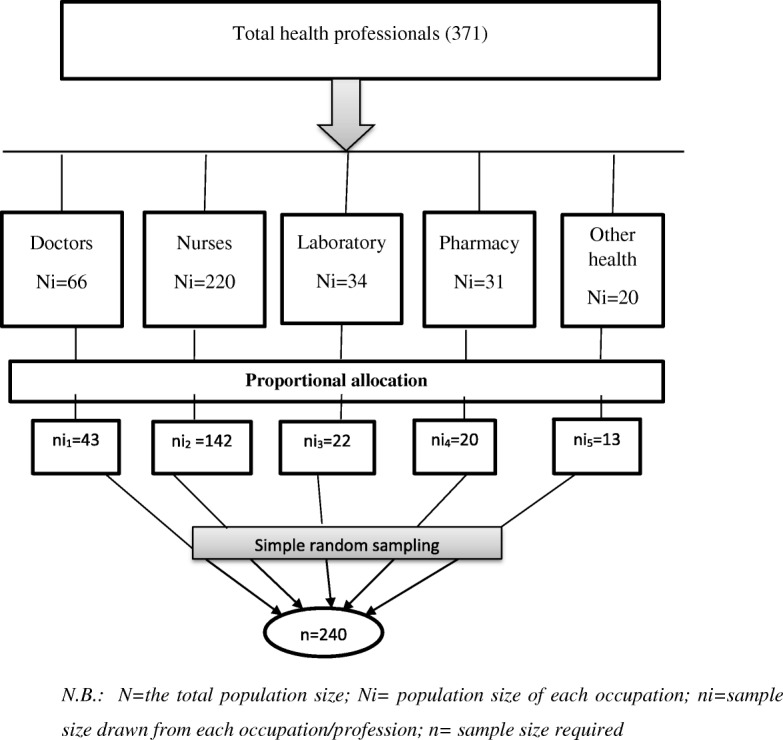
Schematic presentation of the sampling technique. N.B.: *N* = the total population size, Ni = population size of each occupation, ni = sample size drawn from each occupation/profession, *n* = sample size required

The sample size was determined by a single population proportion formula using the prevalence of bacterial contamination from a study conducted in Hospital of the University of Gondar (0.98) [6], with a margin of error of 0.03 and *Z* score for 95% confidence interval of 1.96, and finally, a 15% non-response rate was added. The final sample size was 240. This sample size was allocated proportionally to the number of health professionals in the hospital. Then, a simple random sampling technique was used to select the mobile phones of individual health care professionals (Fig. 1).

### Data collection methods

#### Data were collected using

##### Interview

Data were collected by a self-administered questionnaire after oral and written consent obtained from health professionals. The questionnaire was developed from different literature [6, 16, 17] which has two parts. Socio-demographic variables like age, sex, and educational level were the first part, while the second part includes mobile phone-related questions like the type of mobile phone, cleaning habit of a mobile phone, duration of mobile phone use, and the location for mobile use.

##### A swab of mobile phone for microbial culture and identification

After completion of self-administered questionnaires, a swab sample was collected from the participant’s mobile phone. Before taking a swab, both hands of laboratory technicians were cleaned using an alcohol-based instant hand sanitizer, and powder-free disposable gloves were worn per sample throughout the work to prevent cross-contamination. Sterilized cotton swab moisten by sterile normal saline was rotated to swipe from overall (screen, keypad, sides, and back) area of the mobile phone. In the case of mobile phones with covers, the swab was taken from the outer surfaces of the cover, besides the screen [16]. Then, the mobile phone swab was placed immediately into sterile normal saline in a sterile container and transported to the Microbiology Laboratory, Department of Medical Laboratory Sciences at Haramaya University, within 30 min for microbiological analysis as described by Shooriabi et al. [18].

The collected samples were inoculated onto Blood Agar and MacConkey Agar (Oxoid, LTD, UK) by following the standard streak plate technique [19]. The inoculated plates were incubated aerobically at 37 °C for 24–48 h. Primary isolation of bacteria was made based on their colony characteristics and Gram stain reaction microscopically. Different biochemical tests like triple sugar iron agar, indole, citrate, oxidase, urease, motility, Voges–Proskauer, methyl red, mannitol, catalase, and coagulase were used for further identification.

##### Antimicrobial susceptibility test

Antimicrobial susceptibility test was done according to the Clinical Laboratory Standards Institute guidelines [20] using the Kirby-Bauer disc diffusion method. In brief, the pure isolate (four to five colonies) was added to a sterile tube containing 5 ml of normal saline and mixed gently until it forms a homogeneous suspension. The turbidity of bacterial suspension was standardized by using 0.5 McFarland standards. A sterile cotton swab was dipped into the suspension and inoculated the bacterial suspension over the entire surface of Mueller Hinton agar (Oxoid Ltd., UK) and left at room temperature to dry for 3 to 5 min. Then, antimicrobial drug discs were placed by using a disc dispenser on to the Muller Hinton agar and incubated at 37 °C for 18–24 h. At the end of the incubation period, the diameter zone of inhibition was measured by using a digital caliper. The growth inhibition zone was interpreted as susceptible, intermediate, or resistant after comparison with standard guidelines [20].

### Operational definitions

*Hand hygiene* is a term used to cover both hand washing using soap and water, and cleaning hands with waterless or alcohol-based hand sanitizers.

*Keypad mobile phone* is a mobile phone with the screen installed separately on a push-button phone device for dialing a number.

*Touchscreen mobile phone* is a mobile phone display screen that acts as an input device.

### Data quality assurance

The self-administered questionnaire was pretested on 5% of the sample size at Jugol General Hospital. The study participating health professionals were briefly instructed how to fill out the questionnaire. Training on how to collect swab samples was given to data collectors. Completeness of each questionnaire was checked daily during the data collection period. All culture media were prepared by following the manufacturer’s instructions, and sterility was checked by incubating 5% of the prepared culture media at 37 °C overnight and checked for growth of contaminants. The reference strains *Staphylococcus aureus* (ATCC-25923) and *Escherichia coli* (ATCC-25922) were used to check the quality of culture media and antimicrobial discs. Double data entry was done using EpiData to minimize errors during data entry.

### Data analysis

Data were entered into EpiData version 3.1, cleaned, and exported to Statistical Package for Social Sciences (SPSS) program version 20 for further cleaning and analysis. Descriptive statistics like mean, frequency, and percentage were performed on different variables. The magnitude of mobile bacterial contamination was determined as the proportion of those mobile phone samples reported having bacterial isolates by culture test. Bivariate and multivariate logistic regression was performed to identify factors associated with bacterial contamination. A variable with *p* value ≤ 0.25 in the bivariate analysis was a candidate for the multivariate logistic regression in multivariate analysis. The variables with a *p* value < 0.05 were considered statistically significant.

## Results

### Characteristics of participants

Out of 240 health professionals, 226 participated in this study with the response rate of 94.2%. Fourteen participants refused to give swab samples from their mobile phones and were excluded from the study. The mean age of the study participants was 29.3 (± 5.7) years. Majority of the study participants belonged to the age group of 25–29 (46.9%) were male (53.1%), with educational status of bachelor of science/first degree (71.2%) (Table 1). Participants have been using their mobile phones for a minimum period of 1 month to a maximum of 9 years with a mean duration (± SD) of 2.1 (± 1.4) years.

**Table 1:** Socio-demographic characteristics of health professionals (*n* = 226) at Hiwot Fana Specialized University Hospital, Harar, Eastern Ethiopia, Feb–Mar 2018

Socio-demography characteristics	Number (%)
Age	
20–24	38 (16.8)
25–29	106 (46.9)
30–34	44 (19.5)
≥ 35	38 (16.8)
Gender	
Female	106 (46.9)
Male	120 (53.1)
Level of education	
Diploma	21 (9.3)
Bachelor of Science/first degree	161 (71.2)
Medical doctor	36 (16)
Specialist (in medicine)	8(3.5)
Occupation	
Nurse	133 (58.8)
Laboratory technician/technologist	21 (9.3)
Pharmacy	19 (8.4)
Medical doctor	40 (17.7)
Others*	13 (5.8)

*Health officer, physiotherapy technicians, radiologic technicians, anesthetists, ophthalmologists

### Mobile phones and infection prevention

About 80.5% of participants had a touchscreen type of mobile phone and 61.1% of them had no covers. Majority of the respondents did not wash their hands with soap before touching a patient or after using a mobile phone in the hospital setup. About 28.3% of them only had the regular cleaning habit of their mobile phones, and 64.6% of them used to answer a phone call while attending to patients (Table 2).

**Table 2:** Characteristics on the use of mobile phones and infection prevention of health professionals (*n* = 226) at Hiwot Fana Specialized University Hospital, Harar, Eastern Ethiopia, Feb–Mar 2018

Characteristics	Yes no. (%)	No no. (%)
Mobile phone with a cover (lamination)	88 (38.9)	138 (61.1)
Mobile phone use in the hospital	220 (97.3)	6 (2.7)
Use the same mobile phone at home	213 (94.2)	13 (5.8)
Share mobile phone with colleagues	169 (74.8)	57 (25.2)
Answering phone calls while attending to patients	146 (64.6)	80 (35.4)
Regular mobile phone cleaning	64 (28.3)	162 (71.7)
Think that mobile phones can carry bacteria	181 (80.1)	45 (19.9)
Carry your mobile phone with a material used for patient care	164(72.6)	62 (27.4)
Training on infection prevention	113 (50)	113 (50)
Presence of infection prevention manual in a working area	70 (31)	156 (69)
Wash hands with soap/rub with alcohol after using a mobile phone in the hospital	59 (26.1)	167 (73.9)
Wash hands with soap/rub with alcohol before attending to your patient	53 (23.5)	173 (76.5)

Although the majority (80%) of the study participants believed that cell phones could carry bacteria, yet 97.3% of them use their mobile phones in the hospital setup. More than two thirds (72.1%) of the study participants carry their mobile phones with other materials used for the patient’s care. Half of the study participants did not take any kind of infection prevention training, and 69% of them had no infection prevention manual in their working area.

### Prevalence and type of bacterial isolates

**Fig. 2:**
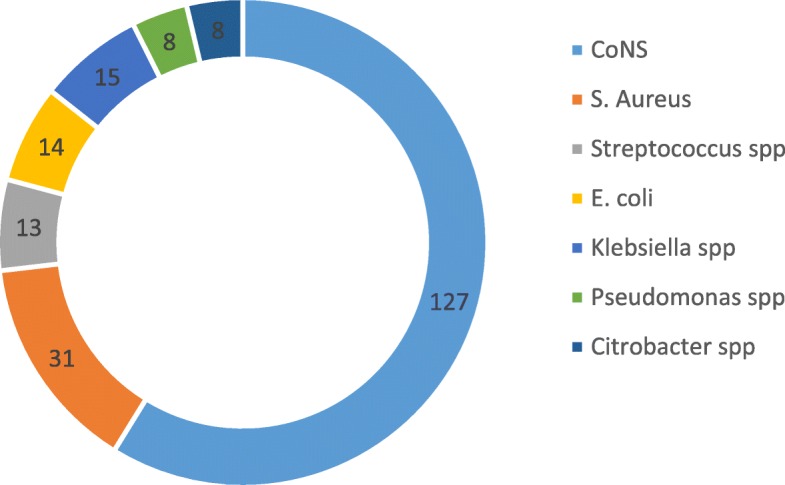
Distribution of bacterial isolates from mobile phones of health professionals at Hiwot Fana Specialized University Hospital, Harar, Eastern Ethiopia, Feb–Mar 2018

The overall prevalence of bacterial contamination amongst the swabbed phone was 94.2% (95% CI 91–97.5). Only three mobile phones showed contamination with multiple bacterial species, and 216 bacterial isolates were identified by phenotypic characterization. Of these bacterial isolates, Gram-positive bacteria (79.2%) were the major isolates, of these, coagulase-negative staphylococci (CoNS) accounted for 58.8% followed by *S. aureus* (14.4%). Amongst Gram-negative bacterial isolates, *Klebsiella* spp. (6.9%) followed by *E. coli* (5.6%) were the main isolates (Fig. 2).

The proportion of mobile phone contamination was higher in the age group of 25–29 years (47.4%). All of the mobile phones carried by laboratory professionals were contaminated with bacterial isolates. The mobile phones owned by 96.8% of the study participants who had not cleaned their mobile phone regularly had bacterial contamination whereas 73% of study participants with no habit of cleaning their hands before attending to the patient had bacterial isolates from the phone. Prevalence of bacterial contamination of phones were about 96.6%, 94.5%, 95.3%, and 94.7% amongst health professionals who answered calls while attending to patients, used their mobile phone in hospital, shared a phone with other colleagues, and had no training on infection prevention.

### Factors associated with mobile phone contamination

In bivariate analysis, male sex, a phone without cover, answering calls while attending to a patient, the absence of regular mobile phone cleaning habit, and lack of hand washing with soap before attending to patients were significant factors selected for multivariate analysis with a *p* value < 0.25.

In multivariate analysis, the incidence of bacterial contamination of mobile phones owned by males was four times higher than that of mobile phones owned by females (AOR 4.1, 95% CI 1.1, 15.8). The incidence of bacterial contamination of mobile phones owned by those health professionals who did not disinfect (clean) phones regularly was also four times more than the incidence of bacterial contamination of mobile phones by those who cleaned their phones regularly (AOR 4.1, 95% CI 1.2, 13.5) (Table 3).

**Table 3:** Factors associated with mobile phone bacterial contamination amongst health professionals (*n* = 226) at Hiwot Fana Specialized University Hospital, Harar, Eastern Ethiopia, Feb–Mar 2018

Characteristics		Mobile phone contaminated				
		Yes (%)	No (%)	Crude OR (95% CI)	Adjusted OR (95% CI)	*P* value
Sex	Male	115 (95.8)	5 (4.2)	4.1 [1.2, 15.5]	4.1 [1.1, 15.8]	
	Female	96 (89.7)	10 (11.3)	1	1	0.041*
Mobile phone with	Cover	128 (92.8)	10 (7.2)	1	1	
	No cover	85 (96.6)	3 (3.4)	2.2 [0.6, 8.3]	1.8 [0.5, 7.2]	0.409
Share phone with colleagues	Yes	161 (95.3)	8 (4.7)	0.5 [0.2, 1.7]		
	No	52 (91.2)	5 (8.7)	1		
Answer calls while attending to patients	Yes	141(96.6)	5 (3.4)	1	1	
	No	72 (90)	8 (10)	0.3 [0.1, 1]	0.4 [0.1, 1.3]	0.129
Regularly clean mobile phone	Yes	56 (87.5)	8 (12.5)	1	1	0.021*
	No	157 (96.9)	5 (3.1)	4.5 [1.4, 14.3]	4.1 [1.2, 13.5]	
Wash hands with soap before attending to patients	Yes	48 (90.6)	5 (9.4)	1	1	
	No	165 (95.4)	8 (4.6)	2.4 [0.7, 6.9]	1.8 [0.5, 6.1]	0.37

*CI* confidence interval, *OR* odds ratio

^a^Sex, the presence of mobile phone cover, answering calls while attending to patients, regular phone cleaning habit, and hand washing with soap before attending to patients were included to calculate the AOR

*Statistically significant at *p* value < 0.05

### Antimicrobial susceptibility pattern of bacterial isolates

As a whole, ceftriaxone (80.6%), ciprofloxacin (77.3%), and gentamicin (72.7%) showed higher activity against bacterial isolates, while ampicillin and trimethoprim-sulfamethoxazole had less effect with a resistance rate of 61.6% and 56.9%, respectively. There was no significant difference in the activity of those drugs against Gram-positive and Gram-negative isolates (Table 4).

**Table 4:** Antimicrobial susceptibility pattern of bacterial isolates from the mobile phones of health professionals (*n* = 226) at Hiwot Fana Specialized University Hospital, Harar, Eastern Ethiopia, Feb–Mar 2018

Bacterial isolates	Total no	Antimicrobial susceptibility *N* (%)								
			AMP	CHL	CRO	CIP	SXT	CN	AMC	ERY
*S. aureus*	31	S	9 (29)	18 (58)	22 (71)	23 (74.2)	8 (25.8)	20 (64.5)	20 (64.5)	19 (61.3)
		I	3 (9.7)	3 (9.7)	3 (9.7)	2 (6.5)	3 (9.7)	4 (12.9)	3 (9.7)	2 (6.5)
		R	19 (61.3)	10 (32.3)	6 (19.3)	6 (19.3)	20 (64.5)	7 (22.6)	8 (25.8)	10 (32.2)
CoNS	127	S	50 (39.4)	86 (67.7)	103 (81.1)	101 (79.5)	40 (31.5)	102 (80.3)	82 (64.5)	78 (61.4)
		I	10 (7.9)	10 (7.9)	9 (7.1)	4 (3.2)	7 (5.5)	1 (0.8)	11 (8.7)	10 (7.9)
		R	67 (52.7)	31 (24.4)	15 (11.8)	22 (17.3)	80 (63)	24 (18.9)	34 (26.8)	39 (30.7)
*Streptococci* spp.	13	S	7 (53.8)	9 (69.2)	12 (92.3)	8 (61.5)	11 (84.6)	1 (7.7)	7 (53.8)	7 (53.8)
		I	–	–	1(7.7)	–	–	1(7.7)	–	–
		R	6 (46.2)	4 (30.8)	–	5 (38.5)	2 (15.4)	11 (84.6)	6 (46.2)	6 (46.2)
		S	3 (21.4)	5 (35.6)	9 (64.3)	12 (85.7)	6 (42.9)	14 (100)	8 (57.1)	9 (64.3)
*E.coli*	14	I	–	1 (14.3)	1 (7.1)	–	–	–	1 (7.1)	1 (7.1)
		R	11 (78.6)	8 (57.1)	4 (28.6)	2 (14.3)	8 (57.1)	–	5 (35.8)	4 (28.6)
		S	9 (60)	10 (66.7)	15 (100)	15 (100)	4 (26.7)	5 (35.7)	9 (60)	8 (53.3)
*Klebsiella* spp.	15	I	–	–	–	–	1 (6.7)	6 (42.9)	2 (13.3)	–
		R	6 (40)	5 (33.3)	–	–	10 (66.6)	4 (21.4)	4 (33.3)	7 (46.7)
*Pseudomonas* spp.	8	S	–	4 (50)	5 (62.5)	3 (37.5)	–	7 (87.5)	–	–
		R	8 (100)	4 (50)	3 (37.5)	5 (62.5)	8 (100)	1 (12.5)	8 (100)	8 (100)
*Citrobacter* spp.	8	S	2 (25)	3 (37.5)	8 (100)	5 (62.5)	3 (37.5)	8 (100)	3 (37.5)	2 (25)
		R	6 (75)	5 (62.5)	–	3 (37.5)	5 (62.5)	–	5 (62.5)	6 (75)
		S	80 (37)	135 (62.5)	174 (80.6)	167 (77.3)	72 (33.3)	157 (72.7)	129 (59.7)	123 (57)
Total		I	13 (6)	14 (6.5)	14 (6.5)	6 (2.8)	11 (5.1)	12 (5.6)	17 (7.9)	13 (6)
		R	123 (56.9)	67 (31.02	28 (13)	43 (19.9)	133 (61.6)	47 (21.8)	70 (32.4)	80 (37)

*AMP* ampicillin, *CHL* chloramphenicol, *CRO* ceftriaxone, *CIP* ciprofloxacin, *CN* gentamicin, *SXT* trimethoprim-sulfamethoxazole, *AMC* amoxicillin-clavulanate, *ERY* erythromycin, *CoNS* coagulase-negative staphylococci species

### Multidrug resistance (MDR) pattern of bacterial isolates

The overall prevalence of MDR bacterial isolates were 69.9%. Amongst all the bacterial isolates, *Pseudomonas* sp. (87.5%), *Klebsiella* sp. (86.7%), and *Citrobacter* sp. (75%) showed MDR characteristics, and *Pseudomonas* sp. exhibited resistance against more than five drugs (Table 5).

**Table 5:** Multiple antimicrobial resistance of bacterial isolates from the mobile phones of health professionals (*n* = 226) at Hiwot Fana Specialized University Hospital, Harar, Eastern Ethiopia, Feb–Mar 2018

	Antibiotic-resistant					
Bacterial isolates	For 2 drugs No. (%)	For 3 drugs No. (%)	For 4 drugs No. (%)	For 5 drugs No. (%)	For 6 drugs No. (%)	For 7 drugs No. (%)
*S. aureus*(*n* = 31)	7 (22.6)	7 (22.6)	3 (9.7)	4 (12.9)	1 (3.2)	–
CoNS (*n* = 127)	30 (23.6)	30 (23.6)	16 (12.6)	6 (4.7)	2 (1.6)	–
*Streptococci* spp. (*n* = 13)	3 (23)	2 (15.4)	2 (15.4)	1 (7.7)	–	–
*E. coli* (*n* = 14*)*	2 (14.3)	1 (7.1)	4 (28.6)	1 (7.1)	–	–
*Klebsiella* spp. (*n* = 15)	6 (40)	4 (26.7)	2 (13.3)	1 (6.7)	–	–
*Pseudomonas* spp. (*n* = 8)	–	–	–	3 (37.5)	3 (37.5)	1 (12.5)
*Citrobacter* spp. (*n* = 8)	–	–	2 (25)	4 (50)	–	–
Total *N* = 216	48 (22.2)	44 (20.4)	30 (13.9)	20 (9.3)	6 (2.8)	1 (0.5)

## Discussion

Mobile phones are widely used in the health care facility as a non-medical device. It has been increasingly used as a means of collecting epidemiological data and monitoring diseases both in the community and in the health care facility [21]. There is no restriction for use of mobile phones in Ethiopia within the health care facilities regardless of their microbial load.

This study revealed that 94.2% of the mobile phones of health professionals were contaminated with bacteria. Similar findings were reported from Hawassa, Ethiopia [22], Gondar, Ethiopia [6], India [23–25], and Iran [26]. However, lower rates of bacterial contamination were also reported from India (24%) [27] and Nigeria (80.6%) [13]. The observed variation might be due to the difference in adherence to infection prevention or frequency of cleaning mobile phones during working hours, hand washing practice, the pattern or policy of mobile use in the hospital, and awareness of health professionals about the role of a mobile phone in microbial transmission.

CoNS (58.8%), *S. aureus* (14.4%), and *Klebsiella* sp. (6.9%) were the predominant isolates. Most studies previously conducted in Ethiopia [6, 17, 28], and outside Ethiopia [13, 26, 29], reported similar bacterial isolates with different isolation rates. However, some other organisms such as *Acinetobacter* sp. and *Micrococci* reported by other studies from India [24, 27] and Belgium [15] were not isolated in the current study.

In this study, CoNS isolates were lower than reports from Iran [23, 29] and higher than a study conducted in Gondar, Ethiopia (47.5%) [6], India (17%) [27], and Egypt (33%). CoNS have relatively low virulence and seem to be a normal flora of the skin; however, it has become increasingly recognized as the most common cause of nosocomial bacteremia associated with indwelling devices [30].

The *S. aureus* isolation rate was in line with two studies conducted in India 18% [31] and 14.07% [24]. Some studies conducted in Ethiopia [6, 17, 28], India [32], Italy (64.1%) [33], and Nigeria (25.6%) [13] reported higher isolation rates. *Klebsiella* sp. (6.9%) was the third predominant bacterial pathogen in this study. This was lower than a study conducted in Belgium (15.25%) [15] and India (19%) [31]. *Escherichia coli* (6.5%) was the fourth bacterial isolate, which is in line with a study conducted in Ethiopia (6.8%) [6] and in Nigeria (5.3%) [13]. However, it was lower than a study conducted in Ethiopia (23.5%) [28], Belgium (25.42%) [15], and India (16%) [31]. The presence of *E. coli* indicates a low level of hand and mobile phone hygienic practice, as the organism is part of the intestinal flora and amongst the leading causes of hospital-acquired infection.

Different factors were associated with contamination of mobile phones. Mobile phones of male health professionals were more contaminated. This is similar to a study conducted in India [34] and Iran [35]. However, this was in contrast to the findings of Pal et al. [24] and Shooriabi et al. [18] which reported no such sex association. The difference might be due to a female’s habit of keeping their mobile phones in a handbag and using phones less frequently in the hospital setup. This is also evident in the present study where most females (66.7%) did not use their mobile in a hospital environment.

Health professionals who did not regularly clean (disinfect) their mobile phone had higher bacterial contamination than those who regularly cleaned their mobile phone. This was supported by other studies [6, 15, 16, 36]; a past study reported a significant decline of mobile phone contamination after treating it with 70% isopropyl alcohol [37]. One previous study concluded that professionals aware of phone contamination did not clean their phones because they were afraid that contact with water or liquid disinfectant might damage the phones [38].

Resistance to one or multiple antimicrobials is the most serious health threats in treating patients [10]. In the present study, CoNS were susceptible to ceftriaxone, ciprofloxacin, and gentamicin while it was resistant to trimethoprim-sulfamethoxazole and ampicillin. This result was similar to other studies conducted elsewhere [6, 24, 39, 40]. *Pseudomonas* sp. showed the highest level of resistance, where all isolates were resistant to ampicillin, trimethoprim-sulfamethoxazole, erythromycin, and amoxicillin-clavulanate. This was consistent with the previous studies conducted in Nigeria [5] and India [24, 27].

Chloramphenicol, ciprofloxacin, ceftriaxone, and gentamycin were effective against most isolates whereas ampicillin and trimethoprim-sulfamethoxazole were not. This finding supports the studies conducted in Ethiopia [22, 40] and Egypt [41] which reported high resistance of bacterial isolates against ampicillin. On the contrary, a higher level of resistance to gentamycin and chloramphenicol was reported by Hadir [41] and Alemu et al. [40], respectively.

MDR bacterial strains could be a result of irrational and unnecessary use of antibiotics [42]. High rates of MDR (69.9%) were reported in the current study. This is in contrast to the findings of Gashaw et al. [6] and Khadka et al. [39] which reported a lower level of multidrug resistance. This proves that mobile phones increase the burden of nosocomial infection unless some mandatory guidelines and measures are taken regarding the use and cleaning of phones in a health care setting. This difference on antimicrobial susceptibility compared to other studies might be due to different bacterial strains, hospital environment, empirical treatment practice, use of antibacterial as a prophylactic, easy availability of some drugs without a prescription, dose of the drug, and indiscriminate/prolonged use of common antibiotics. Some reports suggested a correlation of clonal resistance with empirical usage of antibacterial agents [43]. The stated microorganisms isolated from a surgical site, urinary tract, and other infections with different resistance patterns might have an impact on the health of the patients and the community.

## Limitation of the study

As it is a cross-sectional study, the study did not address the effect of period variations. The small sample size makes it difficult to understand the actual practice of health professionals and to perform further multivariable analysis to identify the effect of specific factors on mobile phone contamination.

## Conclusion

The high prevalence of bacterial contamination from mobile phones of health professionals has been found in this study. More than half of the bacterial isolates were resistant to ampicillin and trimethoprim-sulfamethoxazole, and the majority of bacteria isolates were multidrug resistant. Male in sex and absence of cleaning habit for mobile phones were the significantly associated factors of bacterial contamination of mobile phones in the current study.

Based on the above findings, health professionals should clean their mobile phones after use and wash their hands before and after handling patients in the hospital. It is better to develop and implement the mobile phone use guidelines in the hospital. There is a need for special emphasis on medical health workers and laboratory professionals working in the hospital regarding phone use in the working area and cleaning habit. Ceftriaxone, ciprofloxacin, and gentamicin can be used for the treatment of infected patients with bacteria isolated in this finding in Hiwot Fana Specialized University Hospital. Further studies should be conducted with a large sample size including different possible associated factors and actual practice of health professional.

## Abbreviations

AOR: Adjusted odds ratio
ATCC: American Type Culture Collection
CI: Confidence interval
CoNS: Coagulase-negative staphylococci
HFSUH: Hiwot Fana Specialized University Hospital
MDR: Multidrug resistance
SD: Standard deviation
Spp: Species
IHRERC: Institutional Health Research Ethics and Review Committee
